# Supplementary material for: A Paper-Based Analytical Device for Analysis of Paraquat in Urine and Its Validation with Optical-Based Approaches
Source: Diagnostics (Basel). 2020 Dec 22;11(1):6. doi: 10.3390/diagnostics11010006 (PMC7822122; doi:10.3390/diagnostics11010006)
Supplement: Supplementary file 1 [file diagnostics-11-00006-s001.pdf]

Supplementary information

A paper-based analytical device for analysis of paraquat in urine and its validation with optical-based approaches

Tse-Yao Wang,[1,+] Yi-Tzu Lee,[1,2,+] Hsien-Yi Chen,[3,4] Cheng-Hao Ko,[5,6] Chi-Tsung Hong,[6] Jyun-Wei Wen,[6,7] Tzung-Hai Yen,[8,*] and Chao-Min Cheng[7,*]

[1] Department of Emergency Medicine, Taipei Veterans General Hospital, Taipei, Taiwan

[2] School of Medicine, National Yang-Ming University, Taipei, Taiwan

[3] Department of Emergency Medicine, Chang Gung Memorial Hospital, Taoyuan, Taiwan

[4] College of Medicine, Chang Gung University, Taoyuan, Taiwan

[5] Graduate Institute of Automation and Control, National Taiwan University of Science and Technology, Taipei, Taiwan

[6] Spectrochip Inc., Hsinchu, Taiwan

[7] Institute of Biomedical Engineering, National Tsing Hua University, Hsinchu, Taiwan

[8] Department of Nephrology, Clinical Poison Centre, Kidney Research Centre, Centre for Tissue Engineering, Chang Gung Memorial Hospital and Chang Gung University, Taoyuan, Taiwan

[+]These authors contributed equally to this work.

*Correspondence: m19570@adm.cgmh.org.tw (T.-H.Y.); chaomin@mx.nthu.edu.tw (C.-M.C.)

Received: September 17, 2020

Table S1 Developed paper-based device measurement results for 50 ppm paraquat at different reaction times

ΔRGB*	0 min	5 min	10 min	15 min	20 min
Test 1	48.3559	63.5782	81.6701	50.6486	61.3760
Test 2	37.1812	57.5128	80.0336	48.1173	55.7682
Test 3	49.4987	69.5623	80.9990	54.0720	64.8358
Average	45.0119	63.5511	80.9009	50.9460	60.6600
Standard deviation	6.8056	6.0248	0.8227	2.9884	4.5760

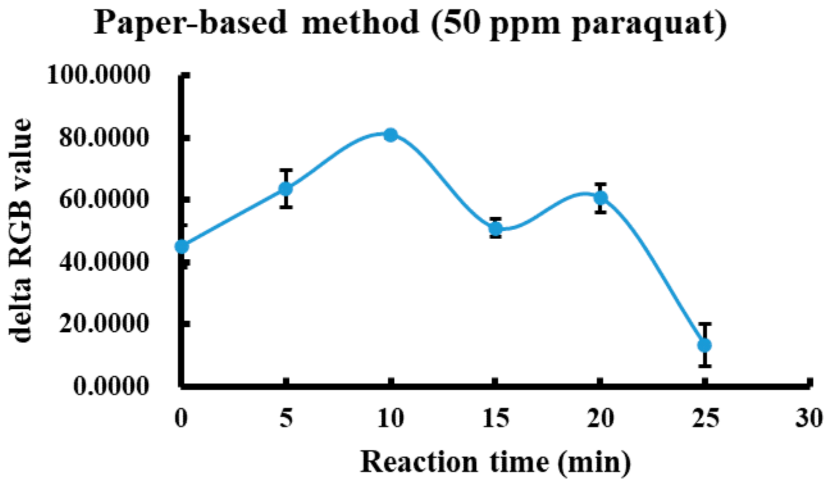

Figure S1. Developed paper-based device measurement results for 50 ppm paraquat at different reaction times.
